# The genome sequence of the Leopard Moth, *Zeuzera pyrina* (Linnaeus, 1761)

**DOI:** 10.12688/wellcomeopenres.19064.1

**Published:** 2023-02-22

**Authors:** Douglas Boyes, University of Oxford and Wytham Woods Genome Acquisition Lab, Darwin Tree of Life Barcoding collective, Wellcome Sanger Institute Tree of Life programme, Wellcome Sanger Institute Scientific Operations: DNA Pipelines collective, Tree of Life Core Informatics collective, Peter W. H. Holland, Darwin Tree of Life Consortium

**Affiliations:** 1 UK Centre for Ecology and Hydrology, Wallingford, Oxfordshire, UK; 2 University of Oxford, Oxford, Oxfordshire, UK

**Keywords:** Zeuzera pyrina, the Leopard Moth, genome sequence, chromosomal, Lepidoptera

## Abstract

We present a genome assembly from an individual male *Zeuzera pyrina* (the Leopard Moth, Arthropoda; Insecta; Lepidoptera; Cossidae). The genome sequence is 687 megabases in span. Most of the assembly is scaffolded into 31 chromosomal pseudomolecules, including the assembled Z sex chromosome. The mitochondrial genome has also been assembled and is 15.3 kilobases in length. Gene annotation of this assembly on Ensembl identified 22,738 protein coding genes.

## Species taxonomy

Eukaryota; Metazoa; Ecdysozoa; Arthropoda; Hexapoda; Insecta; Pterygota; Neoptera; Endopterygota; Lepidoptera; Glossata; Ditrysia; Cossoidea; Cossidae; Zeuzerinae; *Zeuzera*; *Zeuzera pyrina* (Linnaeus, 1761) (NCBI:txid1101072).

## Background

The Leopard Moth *Zeuzera pyrina* is a large (40–60 mm wingspan) moth in the family Cossidae. The common name derives from the ‘leopard-spotted’ pattern of black and grey blotches on white, partly translucent forewings. The species is distributed widely across Europe, with additional scattered records from Russia, the middle East, Japan and North Africa ( GBIF Secretariat, 2022). The moth is also found in eastern Canada and north-eastern regions of the United States where it was accidently introduced in the nineteenth century ( GBIF Secretariat, 2022; Solomon, 1995). In the UK, the species has been recorded across southern counties of England and south-east Wales but is not found in northern counties ( Randle *et al.*, 2019). A record from Scotland in 2022 was almost certainly via a single larva inside a garden shrub transported in the horticultural trade ( Eagleson, 2022). The larvae of *Z. pyrina* bore inside the trunks and branches of living deciduous trees where they live for two or three years, tunnelling and feeding on wood, before pupating underneath the bark. Digestion of lignocellulose seems to be aided by production of cellulase enzymes by bacteria in the larval gut ( Dehghanikhah *et al.*, 2020). The adults do not feed.

The polyphagous wood-boring habit has allowed *Z. pyrina* to reach pest status in many countries, causing damage and yield loss to commercial crops such as nuts, olives and fruit. Examples include damage to olive plantations in Italy and Egypt ( Guario *et al.*, 2002; Hegazi *et al.*, 2015), walnut trees in Iran ( Saeidi *et al.*, 2022) and apple orchards in Greece, Bulgaria and Italy ( Haniotakis *et al.*, 1999; Kutinkova *et al.*, 2006; Pasqualini & Natale, 1999). Control measures that have been attempted include application of insect growth inhibitors, organophosphate pesticides, pheromone traps and entomopathogenic nematodes ( Ashtari *et al.*, 2011; Guario *et al.*, 2002; Salari *et al.*, 2021).

A genome sequence for *Z. pyrina* will be of great interest in understanding the interactions between insects and their bacterial symbionts, and may facilitate development of targeted pest control methods.

## Genome sequence report

**Figure 1.**
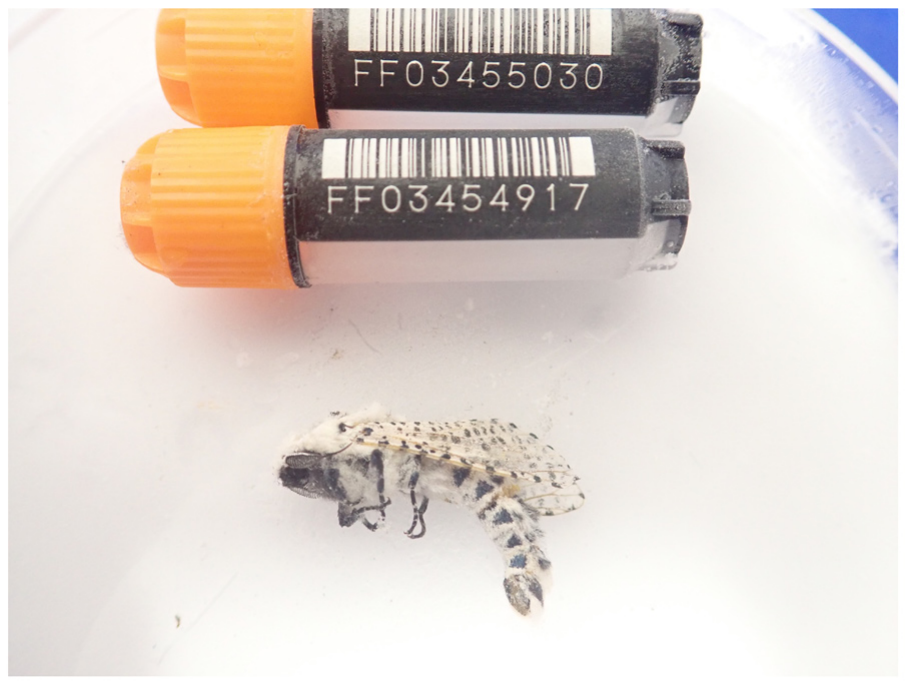
Photograph of the *Zeuzera pyrina* (ilZeuPyri1) specimen used for genome sequencing.

The genome was sequenced from one male *Z. pyrina* specimen ( Figure 1) collected in Wytham Woods, UK (latitude 51.77, longitude –1.33). A total of 38-fold coverage in Pacific Biosciences single-molecule HiFi long reads and 68-fold coverage in 10X Genomics read clouds were generated. Primary assembly contigs were scaffolded with chromosome conformation Hi-C data. Manual assembly curation corrected three missing or mis-joins and removed three haplotypic duplications, reducing the scaffold number by 14.29%.

**Figure 2.**
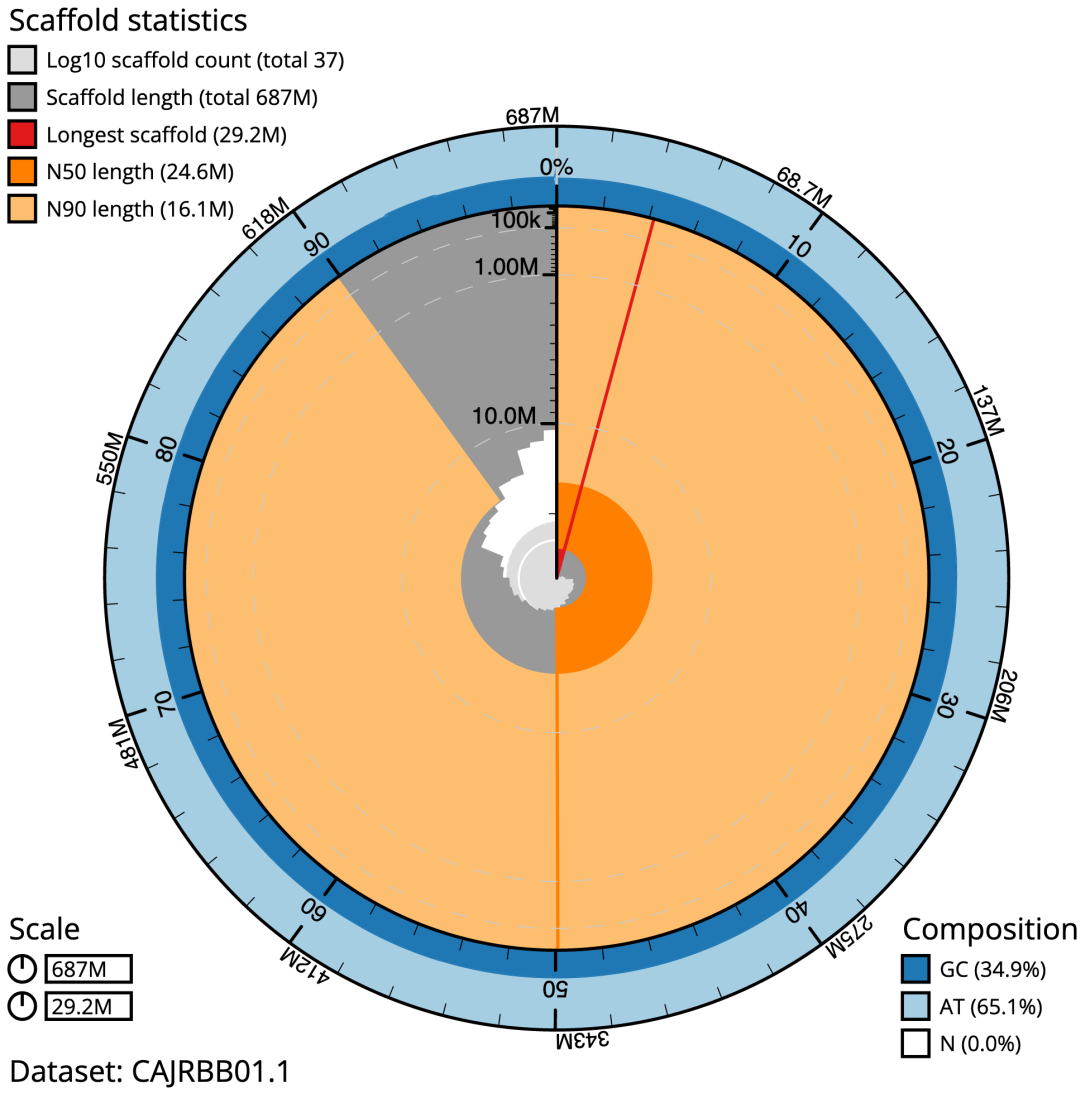
Genome assembly of *Zeuzera pyrina*, ilZeuPyri1.1: metrics. The BlobToolKit Snailplot shows N50 metrics and BUSCO gene completeness. The main plot is divided into 1,000 size-ordered bins around the circumference with each bin representing 0.1% of the 686,903,256 bp assembly. The distribution of scaffold lengths is shown in dark grey with the plot radius scaled to the longest scaffold present in the assembly (29,234,349 bp, shown in red). Orange and pale-orange arcs show the N50 and N90 scaffold lengths (24,575,201 and 16,131,496 bp), respectively. The pale grey spiral shows the cumulative scaffold count on a log scale with white scale lines showing successive orders of magnitude. The blue and pale-blue area around the outside of the plot shows the distribution of GC, AT and N percentages in the same bins as the inner plot. A summary of complete, fragmented, duplicated and missing BUSCO genes in the lepidoptera_odb10 set is shown in the top right. An interactive version of this figure is available at https://blobtoolkit.genomehubs.org/view/ilZeuPyri1.1/dataset/CAJRBB01.1/snail.

The final assembly has a total length of 686.9 Mb in 36 sequence scaffolds with a scaffold N50 of 24.6 Mb ( Table 1). The whole assembly sequence was assigned to 31 chromosomal-level scaffolds, representing 30 autosomes and the Z sex chromosome. Chromosome-scale scaffolds confirmed by the Hi-C data are named in order of size ( Figure 2– Figure 5; Table 2). The assembly has a BUSCO v5.3.2 ( Manni *et al.*, 2021) completeness of 98.7% (single 98.4%, duplicated 0.3%) using the lepidoptera_odb10 reference set. While not fully phased, the assembly deposited is of one haplotype. Contigs corresponding to the second haplotype have also been deposited.

**Table 1.** Genome data for *Zeuzera pyrina*, ilZeuPyri1.1.

Project accession data		
Assembly identifier	ilZeuPyri1.1	
Species	*Zeuzera pyrina*	
Specimen	ilZeuPyri1	
NCBI taxonomy ID	1101072	
BioProject	PRJEB44835	
BioSample ID	SAMEA7701286	
Isolate information	male: ilZeuPyri1; abdomen (PacBio and 10X), head and thorax (Hi-C) ilZeuPyri2 (RNA-Seq)	
Ass embly metrics *		*Benchmark*
Consensus quality (QV)	62	*≥ 50*
*k*-mer completeness	100%	*≥ 95%*
BUSCO **	C:98.7%[S:98.4%,D:0.3%], F:0.2%,M:1.0%,n:5286	*C ≥ 95%*
Percentage of assembly mapped to chromosomes	100%	*≥ 95%*
Sex chromosomes	Z chromosomes	*localised homologous pairs*
Organelles	Mitochondrial genome assembled	*complete single alleles*
Raw data accessions		
PacificBiosciences SEQUEL II	ERR6454727	
10X Genomics Illumina	ERR6054717–ERR6054720	
Hi-C Illumina	ERR6054721	
PolyA RNA-Seq Illumina	ERR9434974	
Genome assembly		
Assembly accession	GCA_907165235.1	
*Accession of alternate haplotype*	GCA_907165255.1	
Span (Mb)	686.9	
Number of contigs	46	
Contig N50 length (Mb)	23.5	
Number of scaffolds	36	
Scaffold N50 length (Mb)	24.6	
Longest scaffold (Mb)	29.2	
**Genome annotation**		
Number of protein-coding genes	22,738	
Number of gene transcripts	22,892	

* Assembly metric benchmarks are adapted from column VGP-2020 of “Table 1: Proposed standards and metrics for defining genome assembly quality” from ( Rhie *et al.*, 2021). ** BUSCO scores based on the lepidoptera_odb10 BUSCO set using v5.3.2. C = complete [S = single copy, D = duplicated], F = fragmented, M = missing, n = number of orthologues in comparison. A full set of BUSCO scores is available at https://blobtoolkit.genomehubs.org/view/ilZeuPyri1.1/dataset/CAJRBB01.1/busco.

**Figure 3.**
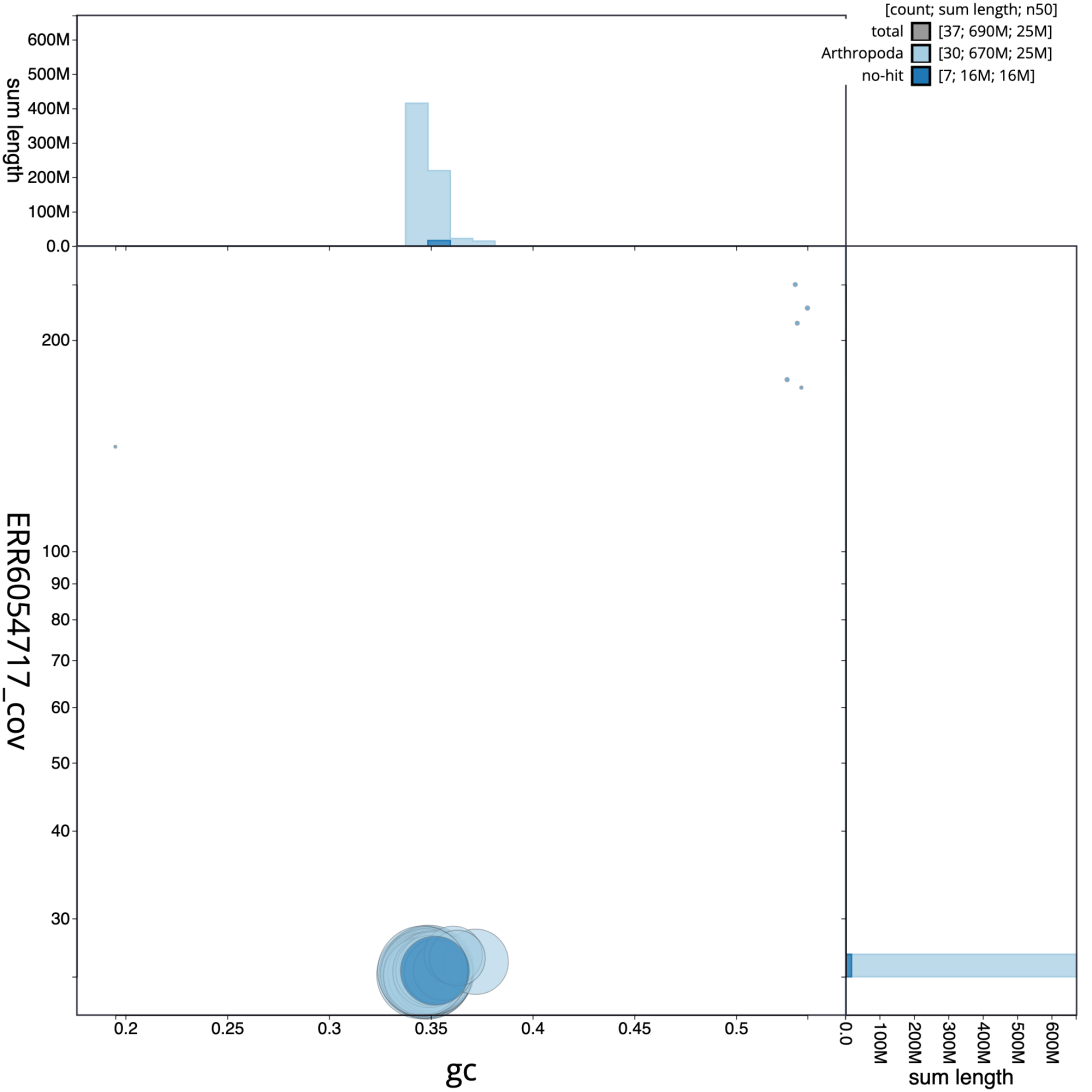
Genome assembly of *Zeuzera pyrina*, ilZeuPyri1.1: GC coverage. BlobToolKit GC-coverage plot. Scaffolds are coloured by phylum. Circles are sized in proportion to scaffold length. Histograms show the distribution of scaffold length sum along each axis. An interactive version of this figure is available at https://blobtoolkit.genomehubs.org/view/ilZeuPyri1.1/dataset/CAJRBB01.1/blob.

**Figure 4.**
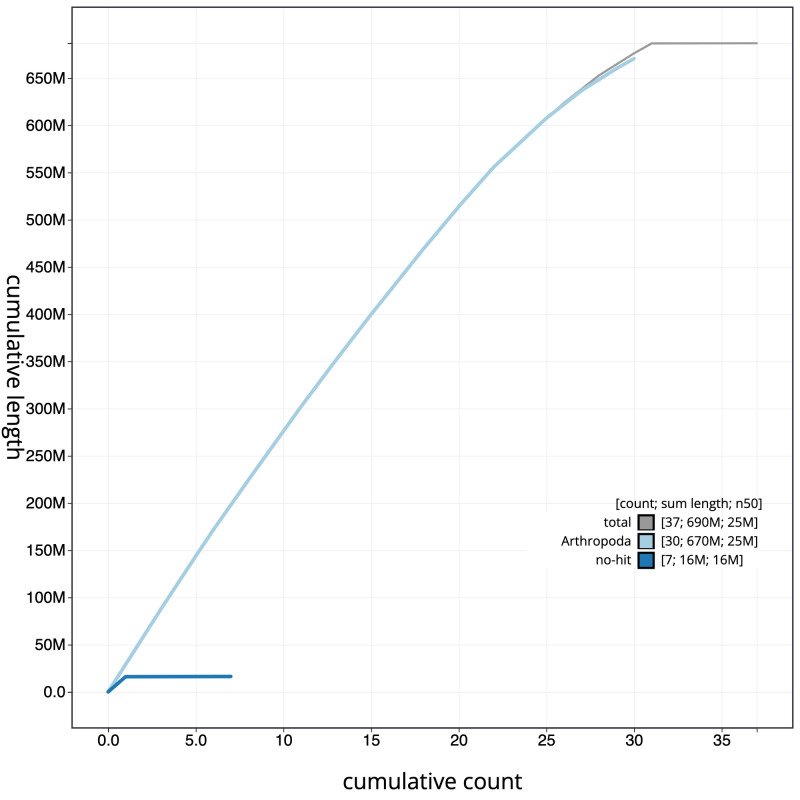
Genome assembly of *Zeuzera pyrina*, ilZeuPyri1.1: cumulative sequence. BlobToolKit cumulative sequence plot. The grey line shows cumulative length for all scaffolds. Coloured lines show cumulative lengths of scaffolds assigned to each phylum using the buscogenes taxrule. An interactive version of this figure is available at https://blobtoolkit.genomehubs.org/view/ilZeuPyri1.1/dataset/CAJRBB01.1/cumulative.

**Figure 5.**
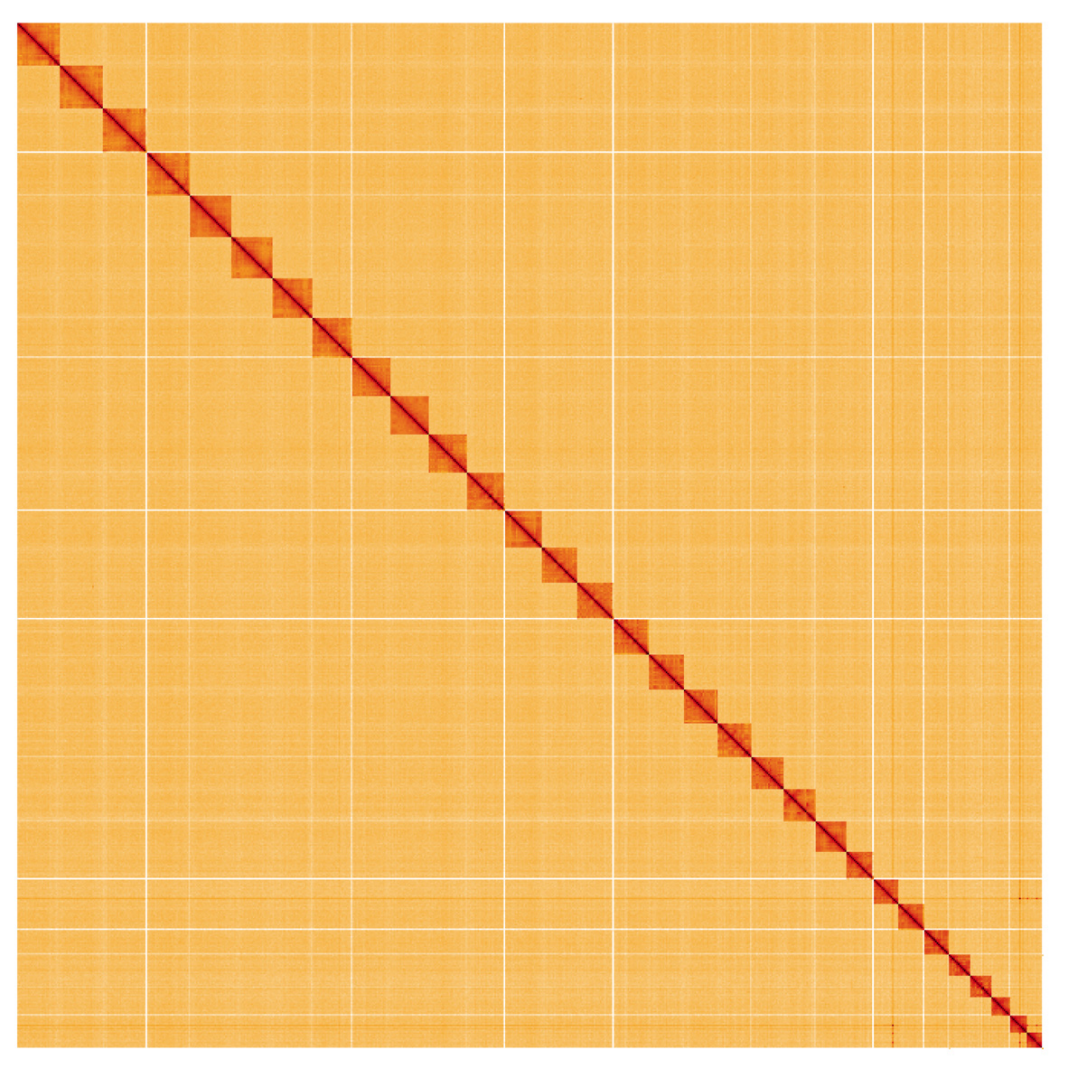
Genome assembly of *Zeuzera pyrina*, ilZeuPyri1.1: Hi-C contact map. Hi-C contact map of the ilZeuPyri1.1 assembly, visualised using HiGlass. Chromosomes are shown in order of size from left to right and top to bottom. An interactive version of this figure may be viewed at https://genome-note-higlass.tol.sanger.ac.uk/l/?d=br9HVfEoSM-9LbyeJXfSQQ.

**Table 2.** Chromosomal pseudomolecules in the genome assembly of *Zeuzera pyrina*, ilZeuPyri1.

INSDC accession	Chromosome	Size (Mb)	GC%
OU015617.1	1	29.23	34.8
OU015618.1	2	29.07	34.8
OU015619.1	3	28.98	34.8
OU015620.1	4	28.48	34.9
OU015622.1	5	27.75	34.7
OU015623.1	6	26.51	34.5
OU015624.1	7	26.24	34.6
OU015625.1	8	26.07	34.7
OU015626.1	9	26.03	34.7
OU015627.1	10	25.53	34.8
OU015628.1	11	24.98	34.7
OU015629.1	12	24.58	34.6
OU015630.1	13	24.21	34.6
OU015631.1	14	23.92	34.9
OU015632.1	15	23.5	34.7
OU015633.1	16	23.45	34.7
OU015634.1	17	22.85	34.9
OU015635.1	18	22.31	35.1
OU015636.1	19	22.13	35.1
OU015637.1	20	21.26	35.1
OU015638.1	21	20.75	34.6
OU015639.1	22	17.69	34.9
OU015640.1	23	17.14	35.2
OU015641.1	24	16.84	35
OU015642.1	25	16.13	35.2
OU015643.1	26	14.63	35.3
OU015644.1	27	14.51	37.2
OU015645.1	28	11.93	35.6
OU015646.1	29	11.47	36.1
OU015647.1	30	10.49	36.3
OU015621.1	Z	28.04	34.6
OU015648.1	MT	0.02	19.8
-	unplaced	0.18	53.1

## Genome annotation report

The *Z. pyrina* genome assembly GCA_907165235.1 was annotated using the Ensembl rapid annotation pipeline ( Table 1; https://rapid.ensembl.org/Zeuzera_pyrina_GCA_907165235.1/). The resulting annotation includes 22,892 transcribed mRNAs from 22,738 protein-coding genes.

## Methods

### Sample acquisition and nucleic acid extraction

Two *Z. pyrina* specimens (ilZeuPyri1 and ilZeuPyri2) were collected in Wytham Woods, Oxfordshire (biological vice-county: Berkshire), UK (latitude 51.77, longitude –1.33) on 25 June 2020. The specimens were caught in woodland habitat using a light trap. Both specimens were collected and identified by Douglas Boyes (University of Oxford) and snap-frozen on dry ice.

DNA was extracted at the Tree of Life laboratory, Wellcome Sanger Institute (WSI). The ilZeuPyri1 sample was weighed and dissected on dry ice with head and thorax tissue set aside for Hi-C sequencing. Abdomen tissue was disrupted using a Nippi Powermasher fitted with a BioMasher pestle. High molecular weight (HMW) DNA was extracted using the Qiagen MagAttract HMW DNA extraction kit. Low molecular weight DNA was removed from a 20 ng aliquot of extracted DNA using 0.8X AMpure XP purification kit prior to 10X Chromium sequencing; a minimum of 50 ng DNA was submitted for 10X sequencing. HMW DNA was sheared into an average fragment size of 12–20 kb in a Megaruptor 3 system with speed setting 30. Sheared DNA was purified by solid-phase reversible immobilisation using AMPure PB beads with a 1.8X ratio of beads to sample to remove the shorter fragments and concentrate the DNA sample. The concentration of the sheared and purified DNA was assessed using a Nanodrop spectrophotometer and Qubit Fluorometer and Qubit dsDNA High Sensitivity Assay kit. Fragment size distribution was evaluated by running the sample on the FemtoPulse system.

RNA was extracted from abdomen tissue of ilZeuPyri2 in the Tree of Life Laboratory at the WSI using TRIzol, according to the manufacturer’s instructions. RNA was then eluted in 50 μl RNAse-free water and its concentration assessed using a Nanodrop spectrophotometer and Qubit Fluorometer using the Qubit RNA Broad-Range (BR) Assay kit. Analysis of the integrity of the RNA was done using Agilent RNA 6000 Pico Kit and Eukaryotic Total RNA assay.

### Sequencing

Pacific Biosciences HiFi circular consensus and 10X Genomics read cloud DNA sequencing libraries were constructed according to the manufacturers’ instructions. Poly(A) RNA-Seq libraries were constructed using the NEB Ultra II RNA Library Prep kit. DNA and RNA sequencing were performed by the Scientific Operations core at the WSI on Pacific Biosciences SEQUEL II (HiFi) and Illumina NovaSeq 6000 (RNA-Seq and 10X) instruments. Hi-C data were also generated from head and thorax tissue of ilZeuPyri1 using the Arima v2 kit and sequenced on the Illumina NovaSeq 6000 instrument.

### Genome assembly

Assembly was carried out with Hifiasm ( Cheng *et al.*, 2021) and haplotypic duplication was identified and removed with purge_dups ( Guan *et al.*, 2020). One round of polishing was performed by aligning 10X Genomics read data to the assembly with Long Ranger ALIGN, calling variants with freebayes ( Garrison & Marth, 2012). The assembly was then scaffolded with Hi-C data ( Rao *et al.*, 2014) using SALSA2 ( Ghurye *et al.*, 2019). The assembly was checked for contamination and corrected using the gEVAL system ( Chow *et al.*, 2016) as described previously ( Howe *et al.*, 2021). Manual curation was performed using gEVAL, HiGlass ( Kerpedjiev *et al.*, 2018) and Pretext ( Harry, 2022). The mitochondrial genome was assembled using MitoHiFi ( Uliano-Silva *et al.*, 2022), which performed annotation using MitoFinder ( Allio *et al.*, 2020). The genome was analysed and BUSCO scores generated within the BlobToolKit environment ( Challis *et al.*, 2020). Table 3 contains a list of all software tool versions used, where appropriate.

**Table 3.** Software tools and versions used.

Software tool	Version	Source
BlobToolKit	3.5.2	Challis *et al.*, 2020
freebayes	1.3.1-17-gaa2ace8	Garrison & Marth, 2012
gEVAL	N/A	Chow *et al.*, 2016
Hifiasm	00.14-r312	Cheng *et al.*, 2021
HiGlass	1.11.6	Kerpedjiev *et al.*, 2018
Long Ranger ALIGN	2.2.2	https://support.10xgenomics.com/genome-exome/ software/pipelines/latest/advanced/other-pipelines
MitoHiFi	2.11.3	Uliano-Silva *et al.*, 2022
PretextView	0.2	Harry, 2022
purge_dups	1.2.3	Guan *et al.*, 2020
SALSA	2.2	Ghurye *et al.*, 2019

### Genome annotation

The BRAKER2 pipeline ( Brůna *et al.*, 2021) was used in the default protein mode to generate annotation for the *Zeuzera pyrina* assembly (GCA_907165235.1) in Ensembl Rapid Release.

### Ethics and compliance issues

The materials that have contributed to this genome note have been supplied by a Darwin Tree of Life Partner. The submission of materials by a Darwin Tree of Life Partner is subject to the Darwin Tree of Life Project Sampling Code of Practice. By agreeing with and signing up to the Sampling Code of Practice, the Darwin Tree of Life Partner agrees they will meet the legal and ethical requirements and standards set out within this document in respect of all samples acquired for, and supplied to, the Darwin Tree of Life Project. All efforts are undertaken to minimise the suffering of animals used for sequencing. Each transfer of samples is further undertaken according to a Research Collaboration Agreement or Material Transfer Agreement entered into by the Darwin Tree of Life Partner, Genome Research Limited (operating as the Wellcome Sanger Institute), and in some circumstances other Darwin Tree of Life collaborators.

## Data Availability

European Nucleotide Archive: *Zeuzera pyrina* (leopard moth). Accession number PRJEB44835; https://identifiers.org/ena.embl/PRJEB44835. ( Wellcome Sanger Institute, 2021) The genome sequence is released openly for reuse. The *Zeuzera pyrina* genome sequencing initiative is part of the Darwin Tree of Life (DToL) project. All raw sequence data and the assembly have been deposited in INSDC databases. Raw data and assembly accession identifiers are reported in Table 1.

## Author information

Members of the University of Oxford and Wytham Woods Genome Acquisition Lab are listed here: https://doi.org/10.5281/zenodo.4789928.

Members of the Darwin Tree of Life Barcoding collective are listed here: https://doi.org/10.5281/zenodo.4893703.

Members of the Wellcome Sanger Institute Tree of Life programme are listed here: https://doi.org/10.5281/zenodo.4783585.

Members of Wellcome Sanger Institute Scientific Operations: DNA Pipelines collective are listed here: https://doi.org/10.5281/zenodo.4790455.

Members of the Tree of Life Core Informatics collective are listed here: https://doi.org/10.5281/zenodo.5013541.

Members of the Darwin Tree of Life Consortium are listed here: https://doi.org/10.5281/zenodo.4783558.
