# Supplementary material for: The genome sequence of the Leopard Moth, Zeuzera pyrina (Linnaeus, 1761)
Source: Wellcome Open Res. 2023 Feb 22;8:94. [Version 1] doi: 10.12688/wellcomeopenres.19064.1 (PMC10643876; doi:10.12688/wellcomeopenres.19064.1)
Supplement: Peer review history

# Reviewer report: Reviewer response for version 1

**DOI:** 10.21956/wellcomeopenres.21137.r69529

**Published:** 2023-11-13

**Authors:** Markus Friedrich

**Affiliations:** 1 Wayne State University, Michigan, USA

This is a succinct and comprehensive description of the long-read sequencing and chromosome-level assembly of the Leopard Moth, *Zeuzera pyrina* genome. Sampling, sequencing, assembly, assembly assessment, and data depositories have been accessibly documented.

 The only issue I noted was in the third sentence of the "Genome sequence report" section where I wasn't sure what the authors meant by "...three missing or mis-joins..."

Are sufficient details of methods and materials provided to allow replication by others?

Yes

Is the rationale for creating the dataset(s) clearly described?

Yes

Are the datasets clearly presented in a useable and accessible format?

Yes

Are the protocols appropriate and is the work technically sound?

Yes

Reviewer Expertise:

Gene family evolution, Comparative Genomics, Insects

I confirm that I have read this submission and believe that I have an appropriate level of expertise to confirm that it is of an acceptable scientific standard.

# Reviewer report: Reviewer response for version 1

**DOI:** 10.21956/wellcomeopenres.21137.r58889

**Published:** 2023-06-06

**Authors:** Lars Höök

**Affiliations:** 1 Uppsala University, Uppsala, Sweden

The data note presents the genome assembly of the Leopard moth, *Zeuzera pyrina*. The species has a relatively large genome of 687 mb, assembled into 31 chromosome-sized scaffolds.

 Production of the assembly is well motivated and will benefit several research areas.

 All steps of the methods are explained in sufficient detail for reproducibility and use appropriate protocols and software.

Are sufficient details of methods and materials provided to allow replication by others?

Yes

Is the rationale for creating the dataset(s) clearly described?

Yes

Are the datasets clearly presented in a useable and accessible format?

Yes

Are the protocols appropriate and is the work technically sound?

Yes

Reviewer Expertise:

Evolutionary genetics, Sex chromosome evolution

I confirm that I have read this submission and believe that I have an appropriate level of expertise to confirm that it is of an acceptable scientific standard.
